# Recognizing the ethical implications of stem cell research: A call for broadening the scope

**DOI:** 10.1016/j.stemcr.2021.05.021

**Published:** 2021-07-01

**Authors:** Lars S. Assen, Karin R. Jongsma, Rosario Isasi, Marianna A. Tryfonidou, Annelien L. Bredenoord

**Affiliations:** 1 Julius Center for Health Sciences and Primary Care, Department of Medical Humanities, University Medical Center Utrecht, 3508 GA Utrecht, the Netherlands; 2 Dr. John T. Macdonald Foundation Department of Human Genetics and John P. Hussman Institute for Human Genomics, University of Miami Miller School of Medicine, Miami, FL 3310, USA; 3 Department of Clinical Sciences, Faculty of Veterinary Medicine, Utrecht University, 3584 CM Utrecht, the Netherlands

**Keywords:** stem cells, ethics, hard and soft impacts, iPSCs, regenerative medicine

## Abstract

The ethical implications of stem cell research are often described in terms of risks, side effects, safety, and therapeutic value, which are examples of so-called hard impacts. Hard impacts are typically measurable and quantifiable. To understand the broader spectrum of ethical implications of stem cell research on science and society, it is equally important to recognize soft impacts. Soft impacts are the effects on behavior, experiences, actions, moral values, and social structures; these are often indirect effects of stem cell research. The combined notions of hard and soft impacts offer a broader way of thinking about the social and ethical implications of stem cell research and can help to steer stem cell research into a sociable desirable direction. Soft impacts enable researchers to become more aware of the broad range of significant implications involved in their work and deserve equal attention for understanding such ethical and societal effects of stem cell research.

## Teaser

The ethical implications of stem cell research are often discussed in terms of risks, side effects, and safety, which are examples of hard impacts. In this article, Assen and colleagues argue that to understand the broader spectrum of ethical implications of stem cell research on science and society, it is important to recognize the so-called soft impacts.

## Main text

### Introduction

Stem cell science has expanded in the past two decades. These new research possibilities raise ethical and policy questions. While ethical reflections on embryonic stem cells have strongly focused on the moral status of the embryo, this is not the case with induced pluripotent stem cells (iPSCs) and adult stem cells. Ethical reflections surrounding these types of stem cells focus primarily on risks of stem cell interventions, what kind of harm unproven stem cell interventions could cause, how to seek informed consent of patients, and questions about ownership (Andrews et al., 2015; Hyun, 2010; King and Perrin, 2014; MacPherson and Kimmelman, 2019). However, stem cell research has other important ethical implications that are easily overlooked.

For example, between 2013 and 2014, clinical researchers conducted a first-in-human study with a mix of allogeneic mesenchymal stem cells and autologous chondrons as an intervention for stimulating autologous cartilage repair in the knee, with promising results (de Windt et al., 2017). During the clinical trial, there were drawbacks in the recovery of some participants. They did not adhere to the instructions of the researchers to be careful with burdening their knee too much, which inadvertently negatively influenced their rehabilitation process. Possibly, some of the patients believed that the stem cell intervention was more effective than it really was. The drawback was not a direct effect of the stem cell intervention itself. It was an effect of how the stem cell intervention affected patient’s beliefs about the therapeutic value that resulted in an undesirable and unforeseen effect of this intervention. Such a mistaken belief in which the research participant overestimates the benefits of the intervention is often referred to as therapeutic misestimation (Habets et al., 2016; Horng and Grady, 2003). This belief can have several causes; it could, for example, have been a result of the positive portrayal of stem cell research in the media (Caulfield et al., 2016). The researchers of the aforementioned study adhered to ethical guidelines, including approval by the Dutch Central Committee on Research Involving Human Subjects, proper informed consent procedures, and taking preventive measures to minimize or mitigate possible harm (de Windt et al., 2017). Despite good preparations and preventive measures, the drawback in recovery was undesirable and, in hindsight, to some extent avoidable. In subsequent studies, researchers and physical therapists used the described example to stress to patients the importance of being careful with mobilizing their knee after surgery.

This example indicates that the existing narrow view of ethical considerations fails to do justice to all ethical implications related to the use and integration of stem cells in society. This view focuses primarily upon issues, such as the harm of unproven stem cell interventions, and side effects, such as teratoma formation, storage of donated tissue, and discussions about ownership (Andrews et al., 2015; Hyun, 2010; King and Perrin, 2014; MacPherson and Kimmelman, 2019). Stem cell research could benefit from a broader conception of ethical considerations, which could contribute to developing effective strategies to enhance the benefits of stem cells and mitigate undesirable effects. This broader conception of ethical implications can be promoted by distinguishing between the narrow view as “hard impacts,” and a type of ethical considerations that is now often being overlooked, referred to as “soft impacts” (Swierstra, 2015; Swierstra and te Molder, 2012; van der Burg, 2009). The terms hard and soft do not refer to the severity of the impact, but to what is actually impacted.

Hard impacts are characterized by two aspects (Swierstra, 2015). First, there is a causal physical relationship between the research, intervention, or technology, and the effect it has. For example, how a drug (technology) improves the health (the effect), or how a drug leads to an undesirable side effect. Second, the research or technology outcome is quantifiable and measurable, such as the gravity of an immune response, the type of gene-expression pattern of stem cell lines (Scudellari, 2016), and the costs to clinically translate stem cell research (Neofytou et al., 2015). These outcomes could, for instance, indicate an increase or decrease in harm. In other words, hard impacts are direct (physical) outcomes or financial effects of the research, technology, or intervention. It often includes risks, side effects, costs, safety, and therapeutic value. These impacts can be both positive and negative for individuals and society.

Soft impacts are characterized by how technologies, research, or interventions affect experiences, perceptions, actions, social structures, and/or moral values, and are therefore not easily quantifiable or measurable (van der Burg, 2009). In that respect soft impacts are often about the psychological and social effects of research and technology. Compared with hard impacts, soft impacts are outcomes that are an indirect effect of research or technology. An overview of potential hard and soft impacts can be found in Table 1.

**Table 1:** Potential hard and soft impacts of stem cell research and stem cell-based interventions

Potential hard impacts: *direct (physical) and measurable outcomes or financial effects of research, technology or interventions*	Potential soft impacts: *indirect effects of research, technology, and interventions on social structures, psychology, and morality. Often difficult to quantify or measure*
• Risk-benefit analysis • Reduction of animals in studies • Therapeutic value of stem cell-based interventions • Storage of (donated) tissue: *e.g., financial costs of storage.* • Ownership of human pluripotent stem cells • Informed consent: *e.g., the documentation of the understanding and approval of patients and research participants* • Change in costs: *e.g., when an intervention becomes cheaper or more expensive* • Research integrity: *e.g., falsifying data by manipulating images* • The documentation of the provenance of stem cells • Regeneration of tissue due to stem cell interventions • Physical side-effects or harm: *e.g., teratoma formation in a patient*	• Revaluing animal research • How increasing costs could affect possibilities for solidarity in healthcare • Regulatory arbitrage: *e.g., when interventions are offered in jurisdictions with favorable legislation and/or existing loopholes. This could lead to stem cell tourism* • Regulatory brokerage: *when new regulatory frameworks are based on competitive advantage instead of ethical or scientific values* • Stem cell hype: *e.g., exaggerated claims about the therapeutic potential of stem cells* • Change in moral status: *e.g., when iPSCs could be used to create (human) embryos* • Therapeutic misconception and misestimation: *wrongful understanding of the goal of research and overestimating the benefit of the therapy* • Burden of normality: *the psychological and social effects on identity when a chronically ill patient (suddenly) becomes healthy* • Commercialization of stem cell research: *e.g., stem cell clinic offering interventions that have not been scientifically scrutinized* • Whether and how donors and patients should be recontacted about new genetic discoveries relevant to their health • Shifting perceptions of health and disease: *e.g., when a previously incurable disease becomes curable*

This paper argues that the notion of soft impacts could help stem cell researchers to become more aware of the wider array of ethical implications involved in their work. The combined notions of hard and soft impacts offer a broader way of thinking about the ethical implications of stem cell research and can help to steer stem cell research and innovation into a desirable direction. Therefore, these terms will be used in this paper as a heuristic tool to exemplify the different ways of thinking about ethical implications of stem cell research and interventions. Taking both types of impacts into account could have merits for responsible development, use, and policy of stem cell interventions.

### Hard and soft impacts: Examples

To illustrate the difference of hard and soft impacts of stem cell research, we draw on organoid research as an example and its impacts on personalized medicine, costs, and animal research. An organoid is defined as an *in-vitro*-generated stem cell-derived structure, mimicking the architecture and physiology of intact organs. These organoids can, among others, be derived from iPSCs and adult stem cells and it has been proven to be a suitable model for disease-modeling research (Bredenoord et al., 2017; de Souza, 2018).

A positive hard impact of this type of technology is that it allows for the creation of new types of personalized interventions, with an increased therapeutic value compared with non-personalized interventions, thereby reducing harm. In terms of quality adjusted life years (QALYs), personalized interventions could be cost-effective (Hatz et al., 2014). However, since personalized medicine may lead to an increase in QALYs compared with conventional alternatives, it is likely that overall costs will also increase (Tiriveedhi, 2018). Therefore, the development of organoids for personalized interventions may also increase the overall costs for healthcare. This financial harm is a possible negative hard impact of the success side of this technology.

By focusing merely on the increasing costs of medical research and innovations, one may overlook the soft impacts and how technological developments are embedded in a broader social context. Within this context, organoid research used in personalized medicine could potentially affect the financial sustainability of solidarity-based healthcare systems. An example of solidarity in healthcare is the collective responsibility for paying the costs in healthcare (Ter Meulen and Maarse, 2008). Here, the insured population contributes with a relatively small amount of money that is reserved for paying the total or a (large) part of society's healthcare costs. When organoid research-based innovations indeed lead to considerably increased healthcare costs, it could affect the surrounding system of solidarity and consequentially our attitudes to others.

The differences between hard and soft impacts are as well highlighted in the example of how organoid technology affects animal research. A possible hard impact of organoid research is reduction and/or replacement of animal studies, two of the 3Rs principles (refinement, reduction, and replacement) that contribute to ethical research (Bredenoord et al., 2017). Animal studies have been considered necessary and acceptable—even if controversial—for conducting safety and efficacy studies. Within this context, a conceivable soft impact of organoid technology is that it could affect how animal studies are *perceived*. Taking the 3Rs of animal studies in mind as an ethical ground rule, it is possible that the ethical acceptability of certain animal studies will be assessed differently because of the possibility to test efficacy and safety by means of organoids. Two concepts are relevant here: subsidiarity and proportionality (Jans et al., 2018). Subsidiarity implies that an action is acceptable because that action is the least morally problematic way of performing research. In that light, organoid technology is generally considered less morally problematic than research on experimental animals. Also, the proportionality of animal research is relevant to consider. This refers to the question whether animal research for testing the effectiveness and safety of new therapies is still proportional (Jans et al., 2018). In the past, studies in which harm was inflicted on animals were considered proportional for acquiring insights into the safety and efficacy of interventions. Nowadays, with organoid technology, animal testing could in certain cases be perceived as disproportional, since it may not be necessary to inflict harm on animals for acquiring insights in efficacy and safety. Therefore, the existence of organoid technology can affect the permissibility of using certain animal studies. Important to note is that, while the field is evolving toward animal-free substitutes, organoid studies are often also not completely “animal-free.” This is due to the fact that Matrigel, which is commonly used to provide the cells with a 3D environment in which they can thrive, is derived from mice (Bredenoord et al., 2017).

By considering hard impacts of a technology or intervention we find multiple advantages. Quantifying outcomes and the assessment of directs risks help to develop safety measures to prevent harm to the health and well-being of patients and research participants. Furthermore, it helps to create a picture of the financial costs. However, quantifying diseases, cells, side effects, and costs, is only part of the ethical implications of these interventions, as the above-mentioned examples explicate. A narrow focus on hard impacts alone comes with the risk of ignoring aspects that are important for the success and acceptance of these interventions. The effect of technology co-producing our morality, such as solidarity and the perception of animal research, is often referred to as “techno-moral change” (Swierstra, 2015). Insights into this techno-moral change through considering soft impacts could contribute to dealing with the ethical challenges of stem cell research. Being oblivious to the soft impacts of technologies and interventions means that the personal and societal effects are missed.

### Implications for stem cell research(ers)

Becoming aware of the soft impacts of stem cell research could help researchers to anticipate ethical implications and to develop new skills. As a result, researchers could benefit from soft impacts to positively impact the quality of research; it provides a way of anticipating and understanding the ethical implications of stem cell technologies.

Funding agencies focus increasingly on the social value of research, thereby making it more relevant for researchers to contemplate social value and impact. Soft impacts can help to analyze the social value of research. Focusing on soft impacts enables to not only look at treatment effects on a disease or saving money, but also how the research could potentially improve societal structures and increase social justice. For example, the social value of stem cell research could be that it promotes social justice or helps to empower a group of patients (e.g., destigmatize or physically benefit and enable more participation in society) and helps the target group to flourish.

To better anticipate the ethical dimensions of stem cell research and stem cell-based interventions, we need scientists who recognize both hard and soft impacts. To this end, training or educating in terms of hard and soft impacts could be a tool for recognizing the ethical implications of stem cell research and a step toward contemplating whether to mitigate, prevent, or stimulate certain soft impacts. This could, for instance, be done by creating or implementing courses in biomedical curricula that involve how early patient involvement could be achieved, how the public could be engaged, and what the ethics of biomedical research involve. To prevent that these courses reinforce the focus on hard impacts, ethical training or education should be broadened by reflecting upon how stem cell research affects experiences, perceptions, actions, social structures, and moral values.

Patients can offer valuable insights into how stem cell research could affect perceptions, expectations, and actions. Engaging with patients could give insights into how their disease creates specific drawbacks and expectations. Doing this in an early stage of the research, could aid researchers in preventing the negative and foster the positive impacts in a timely manner (Supple et al., 2015). Courses should address under which conditions early patient involvement is fruitful, how and when this could be implemented in the study design, and which skills are needed to have meaningful interactions with patients.

Similarly, public engagement and science communication could be addressed in curricula or workshops. Ideally, this should lead to interactions and dialogue where there is room for the concerns of the public (Reincke et al., 2020). Such interactions could provide information about possible social and societal implications of stem cell research. Courses should focus upon how such dialogue could be organized and on skills that foster dialogue and lay translation of research.

Furthermore, education about the ethics of biomedical research can stimulate moral awareness by researchers. Using not only factual information but also vignettes and moral scenarios (Swierstra, 2015) can offer insights in how stem cell research could affect social practices, moral values, or social structures. Other possible enabling methods are organizing interventions within research teams and using games and roleplay. These could be embedded in PhD programs and conference workshops. Altogether, these types of activities may promote the moral imagination (Coeckelbergh, 2006) of researchers and students and thereby help them to learn to think about the soft impacts of their work. By doing so, moral imagination could help to understand and anticipate techno-moral change: the way that technology and morality co-shape each other (Swierstra, 2015). It should be noted that educational research about the desired content and design is necessary.

Moreover, the notion of hard and soft impacts establishes a vocabulary and a broader way of looking at and reflecting on implications of stem cell technology. These insights could serve as a starting point for discussions about responsible and desirable stem cell science and what would be needed to create these circumstances.

### Implications for policy and regulation

Regulation clusters a broad range of rules or principles governing and evaluating human behavior, thereby establishing boundaries between what should be considered acceptable or indefensible actions. As regulation is influenced by local historical, socio-cultural, political, and economic factors, assessing the hard and soft impacts in both policy debates and outcomes contributes to the development of robust regulation. By doing so, regulation not only reflects society’s shared moral values, but also truly takes into account the broad range of impacts for individuals, communities, and societies. Thus, focusing solely on hard impacts is too narrow, as other important factors for the responsible development and use of stem cell interventions can be overlooked.

To advance responsible development of stem cell interventions, an important question is whether new rules and legislation for promoting ethically sound research should be implemented or how much leeway organizations and researchers should have to deal with the impacts themselves. Rules and regulation might be helpful for conceptualizing and adherence to responsibilities (Coeckelbergh, 2006). For instance, the ISSCR (International Society for Stem Cell Research) provides guidelines for safety and efficacy studies, and guidelines for the derivation, banking, and distribution of stem cell lines. This already helps to prevent and mitigate certain hard impacts of stem cell research, such as loss of reliable data due to contamination of stem cell lines and privacy issues in biobanking (International Society for Stem Cell Research (ISSCR), 2016). As such, guidelines, rules, and regulations help to allocate accountability for processes or operations to researchers or groups of researchers and establish international standards. However, this approach has its limitations, since guidelines, rules, and regulations tend to focus on moral impacts that are measurable or quantifiable. When soft impacts are framed in guidelines, rules, and regulations, we risk that possible socio-ethical challenges might be overlooked. Therefore, guidelines, rules, and regulations cannot and should not do all the moral work. It is important to articulate and explicate the ethical dimensions in stem cell research, where it could help researchers to make better decisions about how the research could be conducted in a desirable and responsible manner. The latter in turn, could ultimately be translated in improved policies or regulations.

### Concluding remarks

So far, academic literature, policy, and researchers have focused primarily on hard impacts of stem cell research. Ethical reflection on stem cell research and technology could be broadened by focusing on soft impacts as well. While the term “soft” may sound misleading as being insignificant, the soft impacts are influential for the use and acceptance of these technologies and require more academic and regulatory attention. Broadening the scope of ethical reflection has implications for education, policy, and regulation. The challenge is to find a balance between how much freedom and education researchers should have to deal with possible ethical implications themselves and where policy and regulation could be of help.

It should be noted that, while hard and soft impacts are meaningful heuristic tools to broaden the scope of ethical implications one could assess, the distinction between hard and soft impacts is primarily an analytical distinction, and not always crystal clear (Swierstra, 2015). For instance, certain soft impacts could become hard impacts over time. Nonetheless, anticipating both hard and soft impacts could steer research and innovation into a desirable direction.

More importantly, having a more comprehensive understanding of the ethical implications of stem cell research could help researchers and others to think about how to anticipate and thereby possibly prevent or mitigate possible future challenges instead of dealing with ethical challenges once they emerge.
